# Eyelid Malpositions and Ocular Surface Disease: Clinical Correlations and Management Strategies

**DOI:** 10.3390/jcm14238523

**Published:** 2025-12-01

**Authors:** Francesco M. Quaranta Leoni, Nazareno Marabottini, Adriana Iuliano, Diego Strianese, Gustavo Savino

**Affiliations:** 1 Orbital and Adnexal Service, Tiberia Hospital—GVM Care & Research, Via Emilio Praga 26, 00137 Rome, Italy; 2 Department of Translational Medicine, University of Ferrara, 44121 Ferrara, Italy; 3 Department of Oculoplastic Surgery, San Giovanni Addolorata Hospital, 00184 Rome, Italy; 4 Department of Neurosciences, Reproductive and Odontostomatological Sciences, Division of Ophthalmology, School of Medicine, University of Naples “Federico II”, 80131 Naples, Italy; 5 Ocular Oncology Unit, Fondazione Policlinico Universitario A. Gemelli IRCCS, 00168 Rome, Italy; 6 Ophthalmology Department, Università Cattolica del Sacro Cuore (UCSC), 00168 Rome, Italy

**Keywords:** eyelid abnormalities, ocular surface disease, thyroid eye disease, cicatricial entropion, facial nerve palsy

## Abstract

Eyelid retraction, cicatricial entropion, and deformities associated with facial nerve palsy are among the eyelid malpositions most detrimental to the ocular surface, as they cause exposure, tear film instability, inflammation, and potentially significant visual impairment. These conditions present major functional and esthetic challenges, underscoring the need for a clear understanding of their mechanisms and management. A narrative review was conducted using PubMed, MEDLINE, Embase, and Google Scholar to identify English and non-English studies (with English abstracts) addressing eyelid malpositions related to thyroid eye disease, cicatricial processes, and facial nerve palsy. Screening and cross-referencing yielded 115 relevant publications. Studies were excluded if they lacked clinical relevance, did not address the target disorders, involved animals, consisted of insufficient case reports, lacked an English abstract, or were non–peer-reviewed or duplicated. Extracted information included patient demographics, clinical presentations, diagnostic methods, treatments, complications, and outcomes. In thyroid eye disease, eyelid retraction results from adrenergic overstimulation, increased Müller muscle tone, and fibrosis involving the levator–superior rectus complex. Temporary improvement may be achieved with botulinum toxin, corticosteroids, or soft-tissue fillers, whereas sustained correction requires individualized surgical approaches. Cicatricial entropion arises from posterior lamellar contraction caused by inflammatory or iatrogenic injury and is best treated with lamellar repositioning or grafting procedures. In facial nerve palsy, incomplete blinking, punctal malposition, and lacrimal pump dysfunction contribute to tearing and ocular surface instability; management prioritizes corneal protection, eyelid rebalancing, and adjunctive measures such as botulinum toxin or physiotherapy. Across all conditions, tailored, multidisciplinary care is essential to maintain ocular surface integrity, restore eyelid function, and preserve quality of life.

## 1. Introduction

Eyelid retraction, cicatricial entropion, and deformities related to facial nerve palsy (FNP) represent some of the eyelid malpositions most threatening to the ocular surface, as they lead to exposure, tear film instability, inflammation, and potentially severe visual impairment. These disorders present major functional and esthetic challenges, making a precise understanding of their mechanisms and management essential.

Thyroid eye disease (TED) is an autoimmune inflammatory disorder that affects the orbital tissues and eyelids, frequently resulting in proptosis, eyelid retraction, and ocular surface exposure. The prevalence of ocular surface disease (OSD) among TED patients ranges from 65% to 95% [1]. Structural and biochemical alterations, including orbital fibroblast proliferation, increased orbital volume, and abnormal eyelid positioning, contribute to evaporative dry eye and chronic ocular surface inflammation. Elevated tear levels of inflammatory cytokines such as IL-1β, IL-6, and IL-8 in active TED support the role of local immune-mediated damage in ocular surface pathology [2].

Eyelid retraction is the most common and often the earliest clinical sign of TED, affecting more than 90% of patients [3]. It not only contributes to functional morbidity, such as exposure keratopathy and visual discomfort, but also has profound psychosocial consequences due to its impact on facial appearance [3]. Pathophysiologically, eyelid retraction results from a complex multifactorial correlation of sympathetic overactivity, inflammation-induced edema, fibrotic remodeling of the levator palpebrae superioris along with the Müller muscle, and mechanical displacement from proptosis and restrictive strabismus [4,5]. Modern imaging techniques have elucidated the role of levator complex hypertrophy and superior eyelid complex enlargement in lateral scleral exposure and severity of retraction [6].

Recent advances in artificial intelligence-based technologies have enabled automated measurement of eyelid morphology and objective assessment of TED-related retraction and may support diagnostic precision and disease monitoring [7]. Despite these technological developments, management remains challenging and requires a multidisciplinary approach to optimize ocular surface protection, visual function, and cosmetic outcomes.

Beyond TED-associated retraction, cicatricial entropion constitutes another severe threat to ocular surface integrity. Despite meticulous surgical technique, outcomes in advanced cases may be suboptimal and recurrences are common, making long-term follow-up essential to safeguard the ocular surface and maintain visual function.

Similarly, facial nerve palsy (FNP) produces significant ophthalmic morbidity by disrupting both ocular surface homeostasis and periocular mechanics. Because the seventh cranial nerve innervates the orbicularis oculi, frontalis, and periocular musculature, its dysfunction alters blink dynamics, compromises eyelid closure, and impairs the lacrimal pump, leading to exposure, dryness, and inefficient tear distribution.

Collectively, these conditions exemplify how eyelid malpositions can disrupt the delicate equilibrium of the ocular surface. Recognizing their mechanisms, anticipating their complications, and implementing timely, individualized interventions are essential to preventing irreversible surface damage and preserving visual function.

## 2. Methods

A narrative review methodology was employed to synthesize current evidence on eyelid malpositions associated with thyroid eye disease, cicatricial disorders, and facial nerve palsy. A comprehensive literature search was conducted across PubMed, MEDLINE, Embase, and Google Scholar covering the period from January 1990 to September 2025. Search terms included combinations of keywords and MeSH terms related to eyelid retraction, cicatricial entropion, facial nerve palsy, ocular surface disease, pathophysiology, and surgical or non-surgical management. Both English and non-English articles were considered eligible for inclusion, provided that an English abstract was available.

Titles and abstracts were screened independently by the authors to identify studies addressing the etiology, clinical features, diagnostic approaches, and treatment strategies for the targeted eyelid malpositions. Full-text articles were then reviewed, and reference lists of included publications were examined to identify additional relevant sources. This process yielded a total of 115 publications considered suitable for qualitative synthesis.

Exclusion criteria were applied to ensure relevance and methodological rigor. Studies were excluded if they: (1) did not focus on TED–related eyelid retraction, cicatricial entropion, or FNP; (2) lacked clinical or mechanistic relevance; (3) involved animal models; (4) consisted of isolated or insufficient case reports; (5) were published without an English abstract; or (6) were non–peer-reviewed or duplicated across databases.

For each included study, data were extracted on patient demographics, clinical presentation, diagnostic evaluation, underlying mechanisms, therapeutic interventions, complications, and reported outcomes. Findings were synthesized narratively due to heterogeneity in study designs and outcome measures.

## 3. Ocular Surface Implications of Eyelid Retraction in Thyroid Eye Disease

### 3.1. Pathophysiology

The ocular surface in TED is particularly vulnerable to desiccation, epithelial damage, and tear film instability due to mechanical exposure from both upper and lower eyelid retraction, combined with inflammatory and fibrotic changes in periocular tissues. Tear breakup time (BUT) is significantly reduced in advanced disease, confirming tear film instability [8]. Moreover, lacrimal gland dysfunction and elevated tear cytokine levels further exacerbate ocular surface inflammation and dryness [1,2].

Upper eyelid retraction, by increasing palpebral fissure height and corneal exposure, is a major contributor to ocular surface compromise. It leads to enhanced tear evaporation, disruption of tear film homeostasis, and predisposition to exposure keratopathy. Lower eyelid retraction, often caused by fibrosis of the lower eyelid retractors and orbital volume expansion, contributes to inferior scleral show and further exacerbates exposure-related symptoms [9,10].

Clinically, patients present with ocular discomfort, photophobia, and foreign body sensation; in severe cases, superficial punctate keratitis or corneal ulceration may develop. The characteristic “staring” or “startled” appearance also results in considerable psychosocial distress.

Early detection and management of ocular surface involvement are crucial, as inflammatory dry eye can precede overt orbital changes. Conservative therapy includes frequent instillation of low-viscosity artificial tears during the day, high-viscosity gels or ointments at night, and protective strategies such as eyelid taping or ocular shields during sleep to prevent exposure keratopathy [11].

### 3.2. Management of Eyelid Retraction in TED

Eyelid retraction management aims to restore eyelid symmetry, protect the ocular surface, and alleviate exposure-related discomfort. Treatment choice depends on disease activity, severity, and patient-specific factors. Lee et al. highlighted the progressive nature of retraction during active inflammation and its incomplete resolution in most patients, reinforcing the need for both temporary and definitive interventions [12].

Non-surgical therapies are indicated have expanded options for temporizing management in the early or active inflammatory phase of TED or in patients who are poor surgical candidates. Botulinum toxin, corticosteroids, combination therapy, adrenergic agents, and hyaluronic acid injections provide effective, reversible control of eyelid height. Subconjunctival triamcinolone acetonide (TA) injections can reduce inflammation and muscle hypertrophy, effectively decreasing levator thickness and improving eyelid position [13]. TA is most beneficial in the active phase but may induce intraocular-pressure elevation or vascular complications; careful monitoring is essential. Betamethasone may offer comparable or superior efficacy with fewer injections [14].

Botulinum toxin type A (BTX-A) induces temporary chemodenervation of the levator or Müller muscle, producing short-term eyelid lowering in mild retraction. Doses of 2.5–10 U are typically administered transcutaneous or subconjunctival, with effects lasting one to six months [15]. Complications include transient ptosis and diplopia, and repeated treatments are often required.

Hyaluronic acid (HA) fillers provide a minimally invasive option for mild to moderate eyelid retraction or contour asymmetry. Injection (0.2–0.9 mL) at the level of the eyelid retractors lengthens the posterior lamella and adds volume to counteract retraction [16,17,18]. Results are immediate, with minor adverse effects such as transient edema or ecchymosis. HA treatment appears more effective in active disease stages. 

Teprotumumab, an IGF-1 receptor inhibitor, may reduce proptosis, indirectly improving eyelid position [19]. Although not specific for correcting eyelid retraction, improvement of both upper and lower eyelid position following infusions has been described and attributed to decreased inflammatory tone and repositioning of the globe [20,21]. 

Surgery remains the definitive treatment for persistent eyelid retraction once TED has stabilized for at least 6–12 months [22]. Goals include correction of eyelid malposition, restoration of symmetry, and protection of the ocular surface.

**Figure 1:**
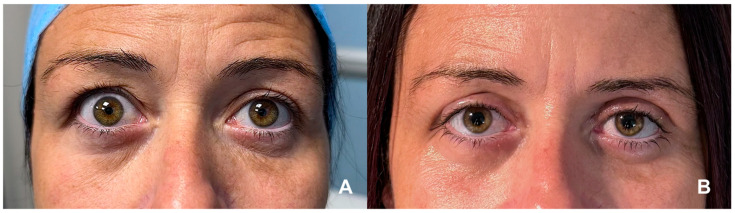
(**A**) Bilateral upper eyelid retraction in a patient with stable TED, with marked scleral show and widened palpebral fissures, more pronounced on the right side. (**B**) Postoperative appearance following right upper eyelid blepharotomy and left Müllerectomy, demonstrating improved eyelid positioning, enhanced symmetry, and reduced ocular surface exposure. (Courtesy of F.M.Q.L.).

For mild retraction (<2.5 mm), posterior approach Müllerectomy or Müller-muscle recession is commonly employed. This technique, performed through a conjunctival incision, excises or recesses Müller’s muscle to achieve controlled lowering. It is effective but may risk under- or over-correction and increase dry-eye symptoms in predisposed patients [23]. Moderate to severe retraction (>2.5 mm) often requires graded anterior levator disinsertion or reinsertion (ALDM) using a transcutaneous approach. The levator aponeurosis is dissected and repositioned to refine eyelid height and contour intra-operatively [24]. In extreme cases, full-thickness blepharotomy or combined techniques may be indicated (Figure 1). Although effective, these carry slightly higher complication rates, including secondary ptosis and possible contour irregularity [25].

When severe proptosis coexists, single-stage orbital decompression with eyelid surgery can be considered, providing both functional and cosmetic benefits while reducing total surgical sessions [26].

Lower eyelid retractor recession, frequently combined with spacer grafts, corrects inferior scleral show and retraction. Common grafts include autologous dermis or hard-palate mucosa (HPM) and alloplastic materials [27]. HPM offers excellent structural support but may become bulky and require revision; dermal grafts are thinner and easier to contour [28]. Orbital decompression performed concurrently with retractor recession can further elevate the lower eyelid by posteriorly repositioning the globe, minimizing the need for secondary surgery [29].

Key surgical considerations include timing (ideally deferred until disease quiescence; early surgery reserved for sight-threatening exposure), material choice (no single spacer material is universally superior—selection depends on patient tissue quality and surgeon expertise) [30], and success rates (77–92% for upper-eyelid surgery, with 8–23% requiring revision) [24,31]. Table 1 summarizes current management strategies for eyelid retraction in TED.

## 4. Cicatricial Entropion and Ocular Surface Considerations

### 4.1. Etiology and Pathophysiology

Cicatricial entropion results from contraction of the posterior lamella (tarso-conjunctival complex) and may involve both upper and lower eyelids. Multiple etiologic factors may contribute to this fibrotic process, including autoimmune, infectious, and traumatic causes. The most frequent are chronic blepharoconjunctivitis, Stevens–Johnson syndrome, ocular cicatricial pemphigoid, drug-induced pseudo–ocular cicatricial pemphigoid, prolonged topical medication use, and trachoma secondary to Chlamydia trachomatis infection [32,33,34].

The most severe forms, potentially leading to blindness, are those associated with Chlamydia trachomatis infection and ocular cicatricial pemphigoid. Ocular cicatricial pemphigoid is an autoimmune disorder with a partially elucidated pathogenesis, characterized by an aberrant immune response to conjunctival epithelial basement membrane antigens. Both entities frequently involve the bulbar conjunctiva and cornea, resulting in significant ocular surface alterations during both the active inflammatory phase and the cicatricial stage. These include misdirected lashes, conjunctival keratinization, and aberrant eyelash growth.

Progressive tarsal fibrosis leads to inward rotation of the eyelid margin, deformity, and vertical shortening of the tarsal plate. Measurement of tarsal height and scleral show (the distance between the limbus and the eyelid margin) in both upper and lower eyelids is essential for clinical assessment. During the active inflammatory phase, topical corticosteroids are frequently used, although the response is often suboptimal. In autoimmune forms, systemic immunomodulatory or immunosuppressive therapies are recommended to control inflammation and limit progression [32,33,34].

### 4.2. Medical Management

Doxycycline, a tetracycline antibiotic, is valuable mainly for its anti-inflammatory and anti-fibrotic properties rather than its antimicrobial effects. It inhibits matrix metalloproteinases (MMPs), which are enzymes involved in collagen breakdown and scar contraction, and suppresses pro-inflammatory cytokines such as interleukin-1 and TNF-α. These mechanisms help limit the fibroblast activity that drives eyelid scarring. Studies on trachomatous trichiasis and other cicatricial eyelid diseases have shown that doxycycline can reduce tissue contraction in vitro and may improve the inflammatory environment around the eyelid margin [35]. Clinically, it is prescribed at 50–100 mg once daily for several weeks before or after surgery to quiet inflammation and reduce recurrence risk. Although doxycycline alone cannot reverse established entropion, its ability to modulate scarring makes it useful in early or mild disease and as preparation for surgery. Side effects such as gastrointestinal discomfort and photosensitivity are generally mild and manageable.

Topical cyclosporine A (0.05–0.1%) acts through a different pathway, inhibiting T-cell activation and cytokine release by blocking calcineurin. In cicatrizing conjunctival disorders like ocular cicatricial pemphigoid and Stevens–Johnson syndrome, chronic inflammation of the conjunctiva and eyelid margin can perpetuate scarring and worsen entropion. Cyclosporine reduces this inflammatory drive and improves tear secretion and surface health. Studies in ocular cicatricial pemphigoid have reported that topical cyclosporine decreases conjunctival inflammation and stabilizes the ocular surface. It is instilled twice daily long-term, often alongside lubricants, to keep the conjunctiva quiet before and after reconstructive surgery. While it cannot reverse fixed scarring, it supports healing and may lower the risk of postoperative recurrence [36].

Both doxycycline and cyclosporine may serve as adjunctive, anti-inflammatory therapies in cicatricial entropion including in combination therapy.

### 4.3. Surgical Options

Despite the wide array of surgical techniques described, management of severe cicatricial entropion remains challenging, with a substantial risk of recurrence. Key parameters for surgical planning include the severity of entropion, degree of eyelid retraction, extent of fornix shortening, margin keratinization, tarsal distortion, and progression of the underlying disease [37,38].

**Figure 2:**
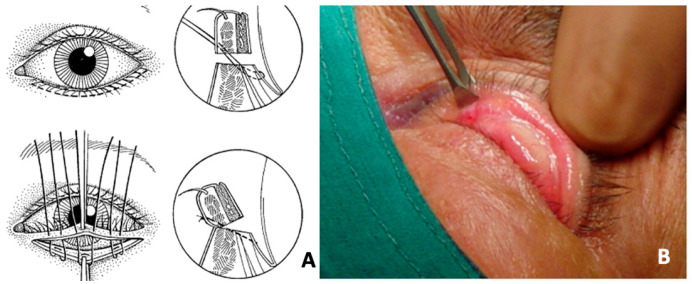
Patient with upper eyelid cicatricial ectropion secondary to drug-induced pseudo–ocular cicatricial pemphigoid. (**A**) Surgical scheme. (**B**) Intraoperative images demonstrating the tarsotomy technique with controlled eversion of the eyelid margin, aimed at releasing cicatricial traction and restoring proper eyelid alignment. (Courtesy of G.S.).

Surgical procedures can be broadly categorized into three main groups: (i)gray-line splitting with anterior lamellar recession and posterior lamellar advancement, (ii) tarsotomy with eyelid margin rotation or eversion (Figure 2), and (iii) posterior lamellar lengthening with grafting [38,39,40,41,42,43]. These may be performed individually or in combination, depending on the severity of fibrosis and anatomical findings.

The gray-line splitting technique with anterior lamellar recession and posterior lamellar advancement is indicated in cases where cicatricial entropion is associated with trichiasis involving the upper and/or lower eyelid [38,39]. The anterior and posterior lamellae are separated along the gray line—posterior to the lash follicles and anterior to the meibomian gland orifices—and deepened by at least 4 mm. In the upper eyelid, this may be combined with a skin-crease incision to achieve full lamellar separation. Detachment and recession of the retractors are often necessary, followed by placement of everting sutures from the superior tarsal border to the skin near the lash line to restore correct lid positioning.

When the tarsal margin is thickened or keratinized, an en bloc excision of 2–3 mm of the anterior lamella (including lash follicles) or a tarsal wedge resection/rotation may be performed [39,40]. In the presence of coexisting trichiasis (lashes arising from the anterior lamella) and distichiasis (lashes arising from the posterior lamella), adjunctive treatments such as cryotherapy, electrolysis, or surgical excision may be indicated [41].

Tarsotomy with rotation or eversion of the eyelid margin remains a widely employed technique for mild to moderate cicatricial entropion affecting either the upper or lower eyelid. A horizontal incision parallel to the eyelid margin is made through the conjunctiva and tarsus approximately 2–3 mm below the lash line. Dissection between the orbicularis muscle and tarsus releases adhesions, after which everting sutures are placed to rotate the eyelid margin outward, restoring normal alignment [41,42].

In severe forms, such as those associated with Stevens–Johnson syndrome, ocular pemphigoid, chemical injury, or advanced trachoma—or in cases of recurrence after previous tarsal fracture or eyelid retraction with scleral show—posterior lamellar grafting is recommended to restore vertical height and reduce ocular surface irritation [32,33,34,42,43]. The mucosal graft is typically secured to the superior edge of the dissected tarsus and the fornix conjunctiva using absorbable sutures. Autologous donor sites include hard palate, buccal mucosa, or tarsoconjunctival tissue, while allogeneic options encompass amniotic membrane and donor sclera. The graft should be slightly oversized in the vertical dimension, with postoperative vertical traction maintained for 48–72 h to minimize contraction and ensure optimal integration [42,43].

### 4.4. Evidence Summary and Outcomes

For an effective approach to cicatricial entropion and to limit ocular surface damage, accurate preoperative assessment, proper staging of the underlying disease, and a tailored surgical plan are essential. Even with meticulous technique, outcomes in severe cases may be limited, and recurrences are frequent. Long-term follow-up is crucial to preserve ocular surface integrity and visual function. Table 2 summarizes the management of cicatricial entropion.

## 5. Oculoplastic Management of Facial Nerve Palsy

### 5.1. Pathophysiology

FNP produces significant ophthalmic morbidity, affecting both ocular surface homeostasis and periocular function [44,45]. The seventh cranial nerve innervates the orbicularis oculi, frontalis, and periocular musculature; its dysfunction disrupts blink dynamics, eyelid closure, and the lacrimal pump, resulting in exposure, dryness, and impaired tear distribution [46]. The primary oculoplastic objectives are preservation of corneal integrity and restoration of eyelid anatomy and dynamic balance [47]. Clinical presentation depends on severity, chronicity, the probability of spontaneous recovery, the presence of synkinesis, and patient-specific functional needs.

Typical features include incomplete or absent blink, lagophthalmos, upper eyelid retraction, paralytic ectropion, brow ptosis, and disordered tear-film dynamics [48]. Denervation leads to orbicularis atrophy and meibomian gland dysfunction, exacerbating evaporative stress. Tear quality may be further diminished by aqueous deficiency in preganglionic lesions and by evaporative instability from poor blink and eyelid malposition; these mechanisms commonly coexist and require individualized management [49].

Several grading systems have been described. The House–Brackmann scale is widely used but lacks ophthalmic specificity [50,51], whereas the Sunnybrook system incorporates dynamic assessment but underrepresents ocular surface compromise. The ophthalmic-specific CADS score (Cornea, Asymmetry, Dynamic function, Synkinesis) emphasizes corneal status and eyelid mechanics and is particularly useful for oculoplastic decision-making [52,53]. These classifications not only stratify severity but also guide timing, with acute cases (<3 months) managed conservatively and persistent paralysis prompting surgical rehabilitation planning [53].

### 5.2. Medical Management

Medical therapy is the cornerstone of acute management, where preventing exposure keratopathy is the priority. Preservative-free lubricants, gels, and ointments are first-line measures, supplemented by avoidance of environmental irritants such as wind or airflow. Moisture chambers or wrap-around shields reduce evaporation, and custom 3D-printed chambers can provide physiologic occlusion when taping is impractical [54,55]. Taping remains a useful nocturnal strategy; hypoallergenic silicone tapes minimize skin trauma, and upper-lid-only splinting can support closure without distorting the lower eyelid [56,57,58]. When lubrication and occlusion are inadequate, temporary tarsorrhaphy provides reliable, short-term corneal protection, particularly following skull-base surgery [59,60].

Specialty devices may further enhance ocular surface stability. Scleral lenses create a continuous fluid reservoir that improves epithelial integrity and vision in exposure states; case series support their safety and benefit in iatrogenic palsy, although availability and tolerance may limit uptake [61,62,63]. Punctal occlusion with temporary plugs or cautery increases tear retention in aqueous-deficient states [64]. Temporary external eyelid weights improve gravity-assisted blink closure and serve as a preoperative trial to titrate the ideal implant weight while maintaining acceptable cosmesis [65,66].

Botulinum toxin has both protective and rehabilitative applications. Injection into the levator palpebrae can induce protective ptosis for three to four months, though transient diplopia may occur with superior rectus spread [67,68]. In synkinesis—such as oral–ocular or paradoxical frontalis activation—targeted pretarsal orbicularis injections reduce involuntary closure, and contralateral chemodenervation improves facial symmetry. Early, low-dose regimens minimize excessive weakening that could worsen ectropion, and tailored treatment improves objective scores and quality of life [69,70].

### 5.3. Surgical Options

Surgical timing is determined by etiology and prognosis. Idiopathic Bell’s palsy typically recovers within approximately six months, warranting conservative or temporary measures. Traumatic or iatrogenic nerve injuries with low recovery potential justify earlier definitive procedures. The development of synkinesis after partial recovery also influences surgical planning.

Upper eyelid loading is generally considered the benchmark technique for managing paralytic lagophthalmos. Gold weights are time-tested, while platinum chains conform more closely to the globe and have lower extrusion and allergy rates [71,72,73]. Segmented platinum chains enable postoperative adjustment for individualized customization [74]. Approaches include transcutaneous skin-crease incisions for direct tarsal fixation—allowing simultaneous levator recession when upper eyelid retraction coexists—and trans-tarsal or transconjunctival placement, which avoids skin incisions and reduces implant prominence [75]. A sutureless posterior transconjunctival technique has also been described [76]. Revision for prominence or migration remains a known requirement and should be discussed preoperatively [77].

Because increased eyelid loading can exacerbate nocturnal lagophthalmos, levator recession with anterior Müllerectomy may be preferable in selected cases. When anterior lamella is deficient, full-thickness skin grafts from the upper eyelid, retroauricular area, or supraclavicular region restore coverage; postoperative function, including CADS scores, improves, although final cosmetic refinement evolves over months [78]. Bupivacaine injection into paretic orbicularis has been reported to improve eyelid closure, lagophthalmos, and epiphora in certain longstanding cases [79].

**Figure 3:**
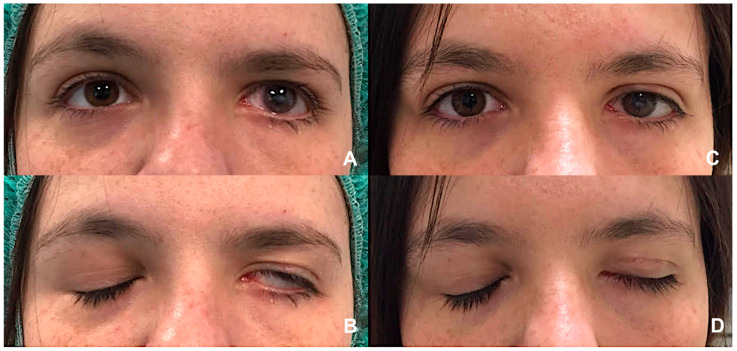
(**A**,**B**) Left lagophthalmos in a patient with longstanding VII nerve palsy, illustrating incomplete eyelid closure and significant exposure of the ocular surface. (**C**,**D**) Postoperative appearance showing improved eyelid closure following gold weight implantation in the left upper eyelid combined with a lateral canthal sling procedure. This combined procedure effectively reduces corneal exposure. (Courtesy N.M.).

Lower eyelid management addresses paralytic ectropion caused by orbicularis weakness, tendon laxity, and vertical deficiency. The lateral tarsal strip procedure provides foundational horizontal tightening (Figure 3). A retrocaruncular medial canthal plication re-establishes medial support without distorting the canthal angle. In cases with horizontal margin deficiency, a lateral periosteal flap canthoplasty recreates a deep lateral tendon and re-establishes physiologic vector orientation, producing durable results [80]. Spacer grafts correct vertical shortage; materials including hard palate mucosa, auricular cartilage, dermis or dermis-fat, and acellular dermal matrix each offer distinct biomechanical advantages [81,82]. Midface lifting elevates descended cheek tissues, redistributes tension, and restores lid–globe apposition, making it especially beneficial in long-standing paralysis with midface descent [83,84]. For severe laxity or recurrent ectropion, autologous fascia lata or temporalis fascia slings provide robust horizontal and vertical stabilization with durable outcomes [85,86].

Lash malposition and cicatricial entropion may occur in chronic palsy due to meibomian gland inversion and tarsal curling. Gray-line split combined with tarsoplasty and anterior lamella repositioning corrects lash misdirection, while selective sphincterotomies release orbicularis contracture and improve corneal protection and esthetics [47,87,88].

Ancillary procedures frequently complement eyelid surgery. Brow ptosis and asymmetry are common, with direct brow lift being suitable for older patients and endoscopic techniques preferred for younger individuals or those with a low hairline. Periosteal fixation enhances long-term stability [89,90,91]. Periorbital volume augmentation with autologous fat or temporary fillers improves symmetry and may help restore blink mechanics [92,93].

Dynamic reanimation techniques—including hypoglossal–facial transfers, cross-face nerve grafts, and free muscle transposition—can restore active blink and periocular tone in selected patients [94,95,96]. These procedures are particularly valuable in younger individuals or those with neurotrophic dry eye, where restoring active closure enhances ocular surface protection [97]. Multidisciplinary collaboration among oculoplastic surgeons, otolaryngologists, neurosurgeons, and peripheral nerve specialists is essential. Specialist-led physiotherapy incorporating neuromuscular retraining, biofeedback, and targeted massage helps prevent maladaptive patterns and improves functional and psychological outcomes [98,99,100,101].

### 5.4. Functional Epiphora

Functional epiphora in FNP refers to symptomatic tearing despite a patent nasolacrimal drainage system. The condition arises from incomplete blink with reduced eyelid excursion, punctal malposition with loss of apposition to the tear lake, and lacrimal pump failure due to orbicularis denervation; these mechanisms frequently coexist with evaporative dry eye, leading patients to paradoxically report both dryness and tearing. Optimal management follows a stepwise approach beginning with restoration of ocular surface homeostasis and punctal apposition, progressing to rehabilitation of the pump mechanism, and reserving bypass procedures for end-stage dysfunction [54].

Assessment should document the temporal pattern of tearing, environmental triggers, and visual fluctuation. Examination includes evaluation of lagophthalmos, Bell’s phenomenon, brow and eyelid position, punctal orientation (capping, eversion, medial canthal laxity), lower-lid distraction and snap-back, and blink symmetry. Fluorescein dye disappearance testing, clearance assessment, irrigation, and cannulation confirm patency and help exclude anatomical obstruction. Endonasal endoscopy, when available, can identify inferior turbinate hypertrophy or mucosal disease interfering with downstream lacrimal function. Symptom scales such as the Munk score provide standardized baselines, and high-speed video blink analysis can quantify closure deficiency and guide rehabilitation.

Initial treatment focuses on stabilizing the ocular surface and reducing reflex tearing. Preservative-free lubricants, gels, and ointments form the foundation of therapy, complemented by environmental modification and moisture chambers or wraparound shields to limit evaporation [54]. Nocturnal occlusion remains valuable; hypoallergenic silicone taping protects the skin, and temporary tarsorrhaphy or botulinum toxin injection into the levator may be required when lubrication and occlusion fail to prevent exposure keratopathy [59,60]. Scleral lenses provide a continuous fluid reservoir that improves epithelial integrity and vision; case series support their safety and benefit in iatrogenic palsy, though tolerance and availability may limit use [61,62,63]. Punctal occlusion with temporary plugs or cautery can increase tear residence time in aqueous-deficient eyes and should be staged carefully to avoid worsening tearing when some pump function remains [64].

Office-based adjuncts can assist before definitive surgery. External adhesive eyelid weights improve gravity-assisted blink closure and function as a preoperative trial to titrate permanent implant weight, often reducing reflex tearing by improving mechanics in primary gaze [65,66]. Long-term symptom control typically requires eyelid rebalancing to restore punctal apposition and lacrimal pump efficiency. Lower eyelid tightening improves globe apposition and reorients the punctum toward the tear lake; punctal repositioning or a limited medial spindle can be added when eversion or scarring predominates. Contemporary systematic reviews support an eyelid-first strategy, showing reduced tearing in eyes without anatomical obstruction and a decreased need for subsequent duct surgery [102,103,104,105]. In practice, combined lateral and medial procedures are often required for maximal pump enhancement.

For persistent epiphora despite optimized eyelid position and surface stability, external or endoscopic dacryocystorhinostomy (DCR) may improve symptoms in selected patients by reducing outflow resistance and enhancing negative-pressure transmission during blink. DCR should complement, rather than replace, eyelid correction, and the endoscopic approach facilitates concurrent intranasal procedures—such as inferior turbinate lateralization—to optimize ostium exposure and long-term patency [102,103,104,105]. When lacrimal pump function is absent despite well-positioned puncta and a stable ocular surface, conjunctivodacryocystorhinostomy (CDCR) with a Lester Jones tube provides a definitive bypass from the conjunctival fornix to the nasal cavity [93,106,107]. Success depends on accurate tube sizing, precise nasal seating, avoidance of septal or turbinate contact, and careful endoscopic guidance. In a facial palsy cohort, symptomatic improvement occurred in approximately 83% initially and 72% at a median of 27.5 months, with extrusion and migration being the most common but manageable complications [108]. Obstruction from debris or biofilm, pyogenic granuloma, and malposition are recognized issues. Rigorous postoperative care—including saline irrigation, routine tube cleaning, and prompt treatment of granulation—improves durability, and patients must receive clear written maintenance instructions and realistic expectations.

Botulinum toxin injection into the palpebral lobe of the lacrimal gland can reduce hypersecretion in crocodile tears syndrome and selected mixed-mechanism epiphora [109]. Cohort and case-series studies show 70–80% response rates with onset within days and effects lasting three to six months; repeat treatments are safe, and adverse effects such as transient ptosis or dry eye are uncommon and self-limited [110,111,112,113,114]. Complete orbital lobe dacryoadenectomy may provide durable relief in carefully selected refractory cases when botulinum toxin A or repeated CDCR are ineffective, contraindicated, or declined, provided the palpebral lobe is preserved to minimize postoperative dry eye [115].

Proximal FNP may also lead to gustatory hyperlacrimation (Bogorad’s syndrome) via aberrant parasympathetic reinnervation, with salivary fibers misdirected through the greater superficial petrosal nerve to the lacrimal gland [109]. Botulinum toxin injection into the palpebral lobe remains the treatment of choice, with 70–80% demonstrating marked improvement within days and effects lasting three to six months; repeat injections are safe, and adverse effects are uncommon and self-limited [110,111,112,113,114].

### 5.5. Evidence Summary and Outcomes

A substantial body of evidence supports the effectiveness of conservative strategies in the acute phase of FNP. Moisture chambers, taping, lubrication, and temporary tarsorrhaphy have consistently demonstrated efficacy in preventing exposure keratopathy [54,56,57,58,59,60]. Specialty scleral lenses have shown benefit in improving epithelial integrity and symptoms in case-series data, although availability and patient tolerance may limit widespread use [61,62,63]. Punctal occlusion is effective in augmenting tear retention in aqueous-deficient states [64], and temporary external eyelid weights offer a reliable method of enhancing blink closure and predicting the outcome of permanent loading procedures [65,66].

A summary of oculoplastic management strategies in facial nerve palsy is provided in Table 3.

## 6. Discussion

Eyelid retraction, cicatricial entropion, and facial nerve palsy share overlapping pathophysiologic mechanisms that compromise eyelid dynamics and ocular surface integrity. Chronic inflammatory and autoimmune processes contribute to fibrosis of the eyelid retractors and posterior lamella, leading to cicatricial entropion, whereas in thyroid eye disease (TED), upper eyelid retraction represents the most prevalent eyelid manifestation. In facial nerve palsy, denervation and imbalance of the periorbital musculature result in lagophthalmos, ectropion, and exposure keratopathy. Collectively, these conditions highlight the delicate balance between eyelid position, muscle tone, and ocular surface protection.

Advances in diagnostic imaging, including AI-based morphometric analysis, have improved the characterization of structural and functional alterations, enhancing preoperative assessment and surgical planning [6,7]. Non-surgical modalities remain valuable adjuncts for modulating inflammation, reducing fibrosis, or providing temporary symptomatic relief, particularly in the early disease phases. However, surgical correction becomes necessary once fibrotic or neuromuscular changes are established.

The trend toward comprehensive, single-stage management—integrating orbital, strabismus, and eyelid procedures—has reduced patient morbidity and healthcare costs while maintaining satisfactory functional outcomes [26]. In parallel, bioengineered grafts and pharmacologic agents targeting fibrotic pathways offer potential for less invasive and more predictable long-term treatment.

In cicatricial entropion, successful correction depends on accurate identification of the underlying etiology, control of active inflammation, and restoration of normal eyelid margin architecture.

In facial nerve palsy, oculoplastic management is best delivered through a stepwise, individualized algorithm encompassing corneal protection, eyelid reanimation, and esthetic balance. Conservative measures—such as lubrication, occlusion, scleral lenses, and temporary tarsorrhaphy—help preserve the ocular surface in the acute phase, whereas chronic cases often require surgical rehabilitation, including upper eyelid loading with platinum chains or levator recession, lower eyelid tightening with spacer grafts, midface lifting, and sling procedures. Ancillary interventions addressing brow position, eyelash direction, and volume deficits further refine symmetry and function. Botulinum toxin serves both protective and rehabilitative purposes, while dynamic reanimation and targeted physiotherapy expand the functional possibilities.

## 7. Conclusions

Eyelid malpositions—particularly eyelid retraction in TED, cicatricial entropion, and paralysis-related deformities—are major contributors to ocular surface morbidity and visual decline. Effective management depends on early recognition, accurate staging, and individualized integration of medical, surgical, and rehabilitative approaches. Achieving optimal outcomes requires meticulous preoperative evaluation, careful control of inflammation, and precise surgical planning.

Several areas of management remain characterized by limited or evolving evidence. The role of teprotumumab in improving TED-related eyelid malpositions is still uncertain; while the drug has demonstrated efficacy in reducing orbital inflammation and soft-tissue expansion, high-quality studies specifically evaluating its impact on eyelid position are lacking, and existing data are derived from small cohorts [19]. Similarly, the choice of spacer graft material for lower eyelid retraction repair remains controversial. A wide range of autologous, allogenic, and synthetic materials has been described, yet most comparative studies are small, retrospective, and heterogeneous, preventing firm conclusions about superiority, long-term stability, or complication profiles [27]. In addition, the timing and sequencing of surgical intervention in patients with partially active TED [26] or evolving cicatricial disease continue to vary among clinicians due to the absence of robust prospective data. These gaps highlight the need for standardized outcome measures and well-designed multicenter studies to better guide evidence-based management.

Despite advances in reconstructive and pharmacologic therapies, outcomes in severe fibrotic or paralytic cases remain limited, and recurrence is a persistent challenge. Long-term follow-up is essential to preserve ocular surface integrity and maintain visual function.

A coordinated multidisciplinary approach involving oculoplastic, corneal, endocrine, neurologic, and immunologic specialists remains central to comprehensive care. Future directions should emphasize minimally invasive, patient-tailored interventions and the development of biocompatible grafts and pharmacologic modulators to enhance postoperative stability and reduce recurrence. The ultimate goal is durable functional restoration, protection of the ocular surface, and improvement in overall quality of life.

## Figures and Tables

**Table 1:** Management Strategies for Eyelid Retraction in TED.

Approach	Treatment/ Procedure	Mechanism of Action/Target	Indication/ Clinical Phase	Advantages/ Outcomes	Limitations/ Complications
**Non-Surgical**	**Subconjunctival** **Triamcinolone** **Acetonide**	Corticosteroid-induced reduction in inflammation and levator hypertrophy	Active inflammatory TED; early retraction	Decreases levator thickness; improves eyelid position	IOP elevation, vascular events; requires monitoring [12]
	**Subconjunctival** **Betamethasone**	Anti-inflammatory corticosteroid effect with longer duration	Active phase TED	Comparable or superior efficacy to TA with fewer injections	Limited long-term data [13]
	**Botulinum Toxin Type A**	Temporary chemodenervation of levator or Müller muscle	Mild retraction; early or transient cases	Minimally invasive; reversible; lasts 1–6 months	Transient ptosis, diplopia; repeated sessions needed [14,15]
	**Hyaluronic Acid Fillers**	Mechanical posterior lamellar lengthening and volumetric expansion	Mild–moderate retraction or contour asymmetry (active phase)	Immediate improvement; outpatient treatment	Transient edema, ecchymosis; temporary duration [16,17,18]
	**Teprotumumab (IGF-1R Inhibitor)**	Reduces orbital inflammation, fibrosis, and proptosis	Active inflammatory TED with eyelid retraction	Improves upper and lower eyelid position indirectly via decreased inflammatory tone	High cost; infusion-related effects [19,20,21]
**Surgical**	**Posterior Approach Müllerectomy/** **Müller Muscle** **Recession**	Partial excision or recession of Müller’s muscle via conjunctival route	Mild retraction (<2.5 mm) in stable phase	Predictable lowering; minimal external scar	Under/overcorrection; worsened dryness [23]
	**Anterior Levator Disinsertion or** **Reinsertion**	Graded disinsertion and reattachment of levator aponeurosis	Moderate–severe retraction (>2.5 mm)	Adjustable intraoperatively; restores symmetry	Secondary ptosis; contour irregularity [24,25]
	**Full-Thickness** **Blepharotomy** **Combined Upper-Lid Techniques**	Complete release of eyelid retractors and anterior lamella	Severe retraction or revision cases	Provides large correction range	Secondary ptosis; contour irregularity; longer recovery [25]
	**Single-Stage Orbital Decompression with Eyelid Surgery**	Concurrent decompression and retraction correction	Severe proptosis with eyelid retraction	Improves globe position and symmetry; reduces staged procedures	Technically complex; higher operative time [26]
	**Lower Eyelid** **Retraction** **Correction (with or without Spacer)**	Lower-lid retractor recession + lengthening of posterior lamella	Stable TED with significant lower eyelid retraction	May incorporate hard-palate mucosa, autologous dermis, or alloplastic spacer materials	Graft-related bulkiness or resorption; may require revision [27,28,29,30]

**Table 2:** Surgical Management of Cicatricial Entropion.

Clinical Problem	Surgical Procedure	Surgical Technique	Advantages/Outcomes	Limitations/ Complications
**Posterior Lamellar** **Contraction** **(Autoimmune,** **Infectious, Traumatic)**	Gray-Line Splitting with Anterior Lamellar Recession and Posterior Advancement	Separation of anterior and posterior lamellae along the gray line; recession of retractors; everting sutures from superior tarsal border to skin near lash line.	Restores eyelid margin alignment; reduces lash-cornea contact.	Possible under- or over-correction; persistent keratinization [38,39].
**Keratinized or** **Thickened Tarsal** **Margin**	Tarsal Margin Excision/Wedge Resection or Rotation	Excision (2–3 mm) of anterior lamella or wedge resection of tarsus; rotation to correct inversion; may include cryotherapy, electrolysis, or lash excision.	Removes abnormal tarsal tissue; re-establishes lid contour.	Margin irregularity, scarring, or recurrence of trichiasis [39,40,41].
**Mild-to-Moderate** **Cicatricial Entropion**	Tarsotomy with Eyelid Margin Rotation/Eversion	Horizontal incision 2–3 mm below lash line through conjunctiva and tarsus; adhesions released; everting sutures rotate the margin outward.	Restores normal margin position; short recovery period.	Limited correction for advanced fibrosis; recurrence possible [40,41,42].
**Severe or Recurrent Cicatricial Entropion** (e.g., Stevens–Johnson, Pemphigoid, Trachoma, Chemical Injury)	Posterior Lamellar Lengthening with Mucosal Graft	Posterior lamella dissected; autologous (hard palate, buccal mucosa) or allogeneic (amniotic membrane, donor sclera) graft sutured to tarsus and fornix.	Restores vertical height; improves ocular surface protection.	Graft contraction or failure; donor-site morbidity; recurrence [32,33,34,36,37,42,43].

**Table 3:** Oculoplastic Management of FNP.

	Procedure	Primary Indication/Objective	Technical Highlights	Advantages	Limitations/ Complications
**Upper Eyelid** **Procedures**	Gold or Platinum Weight Implantation	Paralytic lagophthalmos; incomplete blink closure	Transcutaneous or transconjunctival fixation.	Provides dynamic eyelid closure and corneal protection	Implant prominence, migration, extrusion, allergic reaction [71,72,73,74,75,76,77]
	Levator Recession ± Müllerectomy	Lagophthalmos with upper-lid retraction or nocturnal exposure	Recession of levator aponeurosis ± Müllerectomy	Reduces upper-lid retraction; avoids excessive loading	Risk of postoperative ptosis if overcorrected [78]
	Full-Thickness Skin Grafting	Anterior lamellar deficiency	Autografts from upper eyelid, retroauricular, or supraclavicular donor sites	Restores anterior lamellar coverage and contour	Graft contracture; delayed cosmetic maturation [78]
	Bupivacaine Injection	Chronic palsy with poor orbicularis tone	Targeted injection into paretic orbicularis	Temporary improvement in eyelid closure and tearing	Transient effect; variable response [79]
**Lower Eyelid** **Procedures**	Lateral Tarsal Strip	Paralytic ectropion; horizontal laxity	Shortening and fixation of lateral tarsus to orbital rim	Reliable horizontal tightening and lid reapposition	Possible overcorrection or canthal rounding [80]
	Medial Canthal Plication	Medial canthal laxity	Retrocaruncular approach with tendon plication	Restores medial support while preserving contour	Limited efficacy in severe laxity [80]
	Lateral Periosteal Flap Canthoplasty	Recurrent or severe ectropion	Creation of periosteal flap for canthal reconstruction	Durable, anatomically stable correction	Technically demanding [80]
	Spacer Graft Placement	Vertical lid shortening or retraction	Hard-palate mucosa, auricular cartilage, dermis-fat, or acellular dermal matrix	Restores vertical height and tarsal support	Donor-site morbidity; graft resorption [81,82]
	Midface Lift/ Cheek Elevation	Lid malposition with midface descent	Subperiosteal elevation and fixation	Restores lid–globe apposition and tone	Facial edema; recurrence [83,84]
	Fascia Lata or Temporalis Sling	Severe or recurrent ectropion	Autologous fascial suspension	Provides durable horizontal and vertical stabilization	Harvest-site morbidity [85,86]
**Lash and Lid** **Margin Procedures**	Gray-Line Split and Tarsoplasty	Lash ptosis or cicatricial entropion	Anterior lamellar repositioning with selective sphincterotomy	Redirects lashes; improves corneal protection and cosmesis	Scarring or undercorrection [87,88]
**Brow and** **Periorbital** **Procedures**	Direct or Endoscopic Brow Lift	Brow ptosis or asymmetry	Direct excision or endoscopic elevation with periosteal fixation	Restores symmetry and visual field	Visible scarring (direct) or recurrence [89,90,91]
	Periorbital Volume Augmentation	Periorbital hollowing or asymmetry	Autologous fat transfer or temporary fillers	Improves symmetry and blink mechanics	Reabsorption; contour irregularity [92,93]
**Dynamic** **Reanimation**	Hypoglossal–Facial Transfer/Cross-Face Nerve Graft	Complete facial paralysis	Microsurgical nerve transfer or grafting	Restores active blink and tone	Delayed reinnervation; donor morbidity [94,95,96]
	Free Muscle Transposition	Long-standing paralysis	Free muscle (e.g., gracilis) transfer with neural input	Enables dynamic eyelid and facial movement	Technically complex; variable functional gain [94,95,96,97]
**Lacrimal and Tear-Pump Surgery**	Eyelid Tightening ± Punctal Repositioning	Functional epiphora from pump failure	Combined medial/lateral tightening, medial spindle procedure	Restores punctal apposition and improves pump efficiency	May require adjunct procedures [102,103,104,105]
	External or Endoscopic DCR	Persistent tearing with pump dysfunction	Allows concurrent intranasal correction	Enhances lacrimal outflow	Ostium stenosis; granulation tissue [102,103,104,105]
	CDCR	Complete pump failure	Placement of Lester Jones tube	Provides definitive drainage bypass; long-term symptom relief	Tube extrusion, obstruction, migration, maintenance requirements [106,107,108]
**Adjunctive** **Procedures**	Botulinum Toxin Injection (Lacrimal Gland)	Persistent tearing; crocodile tears syndrome	Injection into palpebral lobe of lacrimal gland	70–80% response rate; minimally invasive	Transient dryness, ptosis; repeat injections required [110,111,112,113,114]
